# Health Problems, Unhealthy Behaviors and Occupational Carcinogens Exposures Among Night Shift Brazilian Workers: Results from National Health Survey, 2019

**DOI:** 10.3390/ijerph22081215

**Published:** 2025-08-01

**Authors:** Fernanda de Albuquerque Melo Nogueira, Giseli Nogueira Damacena, Ubirani Barros Otero, Débora Cristina de Almeida Mariano Bernardino, Christiane Soares Pereira Madeira, Marcia Sarpa, Celia Landmann Szwarcwald

**Affiliations:** 1 Instituto Nacional do Câncer, Coordenação de Prevenção e Vigilância, Área Técnica Ambiente Trabalho e Câncer, Rio de Janeiro 20230-240, Brazil; uotero@inca.gov.br (U.B.O.); debora.bernardino@inca.gov.br (D.C.d.A.M.B.); christiane.pereira@inca.gov.br (C.S.P.M.); marcia.sarpa@inca.gov.br (M.S.); 2 Fundação Oswaldo Cruz, Programa de Pós-Graduação em Informação e Comunicação em Saúde do Instituto de Comunicação e Informação Científica e Tecnológica em Saúde, Rio de Janeiro 20230-240, Brazil; damacenagn@gmail.com (G.N.D.); celia_ls@hotmail.com (C.L.S.)

**Keywords:** survey, night shift work, well-being, lifestyles, occupational carcinogens

## Abstract

Introduction: Night shift work (NSW) has been increasingly addressed in the scientific literature, as it is considered a probable carcinogen. In this study, we investigated the association of NSW with health problems, unhealthy behaviors, and occupational carcinogens. Methods: Cross-sectional study with a sample of 47,953 workers from the 2019 National Health Survey. NSW prevalence was estimated according to sociodemographic characteristics. To investigate the associations of NSW with all study variables, gender stratified logistic regression models were used. The odds-ratio and 95% confidence intervals were estimated. Results: Among men, there was a significant association of NSW with sleep disorders (OR = 1.39; 95% CI: 1.17–1.65), tiredness (OR = 1.68; 95% CI: 1.41–2.00), obesity (OR = 1.41; 95% CI: 1.20–1.66), unhealthy food consumption (OR = 1.28; 95% CI: 1.12–1.46), handling of radioactive material (OR = 2.45; 95% CI: 1.61–3.72), and biological material (OR = 3.18; 95% CI: 3.15–4.80). Among females, NSW was associated with the same variables except obesity, but depressive feelings (OR = 1.35 95% CI: 1.09–1.67), frequent alcohol intake (OR = 1.48; 95% CI: 1.23–1.78), handling of chemical substances (OR = 1.54; OR = 1.54; 95% CI: 1.20–1.97), and passive smoking at work (OR = 1.45; 95% CI: 1.12–1.86) were highly significant. Conclusion: Night shift workers are more vulnerable to occupational carcinogen exposure, experience greater impacts on their well-being, and are more likely to engage in unhealthy behaviors. These findings should be considered in managing and organizing night work in the workplace. Actions to promote healthy work environments should be encouraged to protect workers’ health.

## 1. Introduction

Globally, cancer is the second leading cause of premature death from chronic non-communicable diseases (NCDs). In 2020, data from the World Health Organization indicated that the global burden of cancer contributed to 10 million deaths and 19.3 million new cases in the world population [1]. If cancer mortality continues at this rate of progression, it is estimated that cancer deaths will overtake deaths from cardiovascular diseases to occupy first place in the world ranking [2].

Incidence projections indicate that the global burden of cancer will reach 28.4 million cases in 2040, representing a 47% increase compared to 2020. This increase will be more significant in developing countries (64% to 95%) compared to developed nations (32% to 56%) due to demographic changes related to risk factors associated with globalization and economic growth [3]. Such risk factors are expressed by harmful changes in lifestyle such as low consumption of fruits and vegetables, physical inactivity, smoking and alcohol consumption, as well as by greater exposure to carcinogens in workplaces [4,5]. To date, the International Agency for Research on Cancer (IARC) has identified 79 carcinogens present in workplace environments (physical, chemical, and biological agents), 18 occupational exposure circumstances and 38 types of work-related cancers [6].

An increase in the number of jobs with night shifts, driven by the need to produce goods and provide services continuously and uninterruptedly, as seen in the transport, security, healthcare, and manufacturing sectors, among others, has become essential in a globalized society and in economies that are in constant growth [7]. In Canada, the proportion of workers exposed to night shift work increased by 12% from 1990 to 2011 [8]. In the USA, approximately 25% of the employed population work on shifts that are either rotating, irregular or at night [9]. In 2019, Brazilian data showed that 15.5% of male and 9.4% of female employees worked night shifts [10].

The substantial growth of night shift workers in recent decades has led to the need to expand knowledge about the effects on workers’ health. International research has suggested that the mechanisms involved in the incidence of health problems among night shift workers are related to the dysregulation of the circadian cycle [11] and the reduction in serum melatonin concentrations that affect the levels of hormones involved in glucose and lipid metabolism [12]. In addition, night workers present unhealthy behavior patterns, including higher tobacco and alcohol consumption [13], inadequate eating habits [14], and limited physical activity during leisure-time [15].

Recently, circadian rhythm disturbances have been linked to carcinogenic effects in animal models and the IARC classified night shift work as a probable carcinogen for humans [16] associated with the three most common types of cancer in Brazil in 2023–25: breast, prostate, and colon and rectum cancer [17] (excluding cases of non-melanoma skin cancer). In addition to cancer, several health problems have been associated with night shift work, such as sleep disorders [18], depression [19], cardiovascular diseases [20], type 2 diabetes mellitus [21], obesity [22], among other chronic non-communicable diseases (NCDs).

In Brazil, this topic is still rarely discussed, given the limited number of nationwide studies that have evaluated the association between night work and health implications [10,23].

The objective of this study is to investigate the associations of night shift work with health problems, unhealthy behaviors, and occupational carcinogens in a representative sample of Brazilian night shift workers.

## 2. Methods

This is a cross-sectional study that used data from the National Health Survey (Pesquisa Nacional de Saúde-PNS, in Portuguese) carried out in 2019. The PNS is a nationwide, household-based survey conducted by the Ministry of Health in partnership with the Brazilian Institute of Geography and Statistics (IBGE).

The National Health Survey (PNS) was approved on 23 August 2019, by the National Research Ethics Committee (CONEP), under no. 3,529,376. Fieldwork took place between August 2019 and March 2020. All participants signed the informed consent form authorizing their participation in the survey.

### 2.1. Sampling Design

The PNS is part of the IBGE Integrated Household Survey System and uses a subsample of the IBGE Master Sample. A three-stage cluster sampling was used. In the first stage, the primary selection units (PSUs), composed of one or more census tracts, were randomly selected in each stratum of the IBGE Master Sample. In the second stage, a fixed number of households was randomly selected in each PSU. In the third stage, a household resident aged 15 or older was randomly selected to answer the individual questionnaire. Residents in special census tracts (Indigenous villages, barracks, military bases, penitentiaries, etc.) were excluded from the survey. Since the PNS is a household survey performed by the Brazilian Institute of Geography and Statistics (IBGE) in partnership with the Ministry of Health, the sample is carried out only in permanent private households [24].

A total of 90,846 individuals were interviewed in PNS-2019. The response rate was 93.6%. Further details about the survey, the sampling process, and data weighting are available in previous publications [24].

### 2.2. Study Population

In this study, 47,953 individuals who were employed in the reference week (21–27 July 2019) were considered.

In the PNS, occupation was defined by the following question: “What was your occupation (position or role) in your main job? (defined as the job with the highest number of hours, monthly income, or time spent at work)”. A night shift worker was defined as a person whose main job is between the working hours of 8 pm and 5 am.

### 2.3. Study Variables

Sociodemographic characteristics: gender (male, female), age group (18 to 29 years old, 30 to 39 years old, and 40 to 59 years old), race/skin color (White, mixed race, and Black), level of education (no schooling or incomplete primary education; complete primary education or incomplete secondary education; complete secondary education or incomplete college education; complete college education), per capita income (less than the minimum wage (MW), between 1 and 2 MW, more than 2 MW), household situation (rural/urban).

#### 2.3.1. Health Problems

- Sleep disorders: based on the question of Patient Health Questionaire-9 (PHQ-9) [25].
- “In the last two weeks, how often have you had sleep problems, such as difficulty falling asleep, waking up frequently at night or sleeping more than usual?”
- Tiredness: based on the PHQ-9 question “In the last two weeks, how often have you had problems because you did not feel rested during the day, felt tired or lacked energy?”
- Difficulty concentrating: based on the PHQ-9 question “In the last two weeks, how often have you had trouble concentrating on your usual activities?”
- Depressive feelings: based on the PHQ-9 question “In the last two weeks, how often have you felt depressed, “down” or lacking purpose?”
- Answers based on PHQ9 questions were dichotomized as “No-never or less than half of the days” and “Yes-more than half of the days or almost every day”.
- Obesity (based on self-reported BMI ≥ 30)

#### 2.3.2. Health Behaviors

- Regular consumption of unhealthy foods (defined as the consumption of sweets, soft drinks, juices, or snacks at least five times a week).
- Regular consumption of artificial juices or soft drinks (at least five times a week).
- Often replacing meals (at least five times a week) with snacks.
- Regular consumption of sweets (at least five times a week).
- Currently a smoker (smoking daily or less than daily).
- Alcohol consumption (at least once a week).

#### 2.3.3. Exposure to Possible Occupational Carcinogens

- Handling of radioactive material during transportation, receipt, storage, and X-ray work.
- Handling of chemical substances (pesticides, gasoline, diesel, formaldehyde, lead, mercury, chromium, chemotherapeutic agents).
- Handling of biological material (blood, needles, secretions).
- Passive smoking at work, based on the question: “In the last 30 days, has anyone smoked in the same closed environment where you work?”

### 2.4. Statistical Analysis

Prevalence of night workers and respective 95% confidence interval (95% CI) were estimated according to the categories of each sociodemographic variable. To test differences in work shift (day/night) prevalence by sociodemographic characteristics, a bivariate logistic regression model was used considering each sociodemographic characteristic (gender, age group, level of education, skin color, per capita income, and urban/rural household situation) as the independent variable.

Prevalence (%) of all study variables and respective 95% confidence intervals (95% CI) were estimated according to work shift (day/night). A logistic regression model was used to investigate the associations of the outcome (night shift work) with each independent variable. The odds-ratio (OR) and the 95% confidence interval were used to test the associations at the 5% significance level. The analyses were stratified by gender (male/female).

All statistical analyses were performed using the software SPSS version 21.0, considering the PNS complex sampling design.

## 3. Results

A total of 47,953 workers aged 18–59 years old participated in the study, of whom 6,599 reported working night shifts; 68.2% were males and 31.8% females.

In Table 1, we present the estimated prevalence of night shift workers according to sociodemographic characteristics. Statistically significant differences were observed in the proportion of night shift workers by gender (17.0% among men and 9.7% among women) and by age group, with the highest prevalence of night work in the 30–39 age group (15.1%; 95% CI: 14.1–16.2). There was no statistically significant difference related to skin color. Regarding the level of education, the highest prevalence of night shift work was found among workers who had completed secondary school education. As for income results, the highest percentages of night shift work were found among workers with a per capita income greater than two minimum wages: (17.4%; 95% CI: 16.4–18.5). The proportion of night workers residing in urban areas was higher than in rural areas: 14.6% versus 7.6%, respectively (Table 1).

Prevalence (%) of some emotional problems, unhealthy behaviors, and exposure to possible occupational carcinogens according to work shift and stratified by gender is presented in Table 2, Table 3 and Table 4. There were statistically significant differences between male workers on day and night shifts, in terms of sleep disorders (OR = 1.39; 95% CI: 1.17–1.65) and tiredness (OR = 1.68; 95% CI: 1.41–2.00). Among females, significant differences were found for sleep disorders (OR = 1.23; 95% CI: 1.03–1.46), tiredness (OR = 1.29; 95% CI: 1.08–1.54), and depressive feelings (OR = 1.35; 95% CI: 1.09–1.67).

A positive association of obesity (based on self-reported BMI ≥ 30) with night shift work was found for males (OR = 1.41; 95% CI: 1.20–1.66) as shown in Table 2.

Table 3 shows the association of night shift work and unhealthy behaviors. For males, night shift work (compared to day shift work) was significantly associated with regular consumption of unhealthy foods (OR = 1.28; 95% CI: 1.12–1.46), replacing meals with snacks (OR = 2.52; 95% CI: 1.66–3.83); regular consumption of sweets (OR = 1.23; 95% CI: 1.04–1.45); and alcohol intake at least once a week (OR 1.15; 95% CI: 1.02–1.29). Among females, night workers were more likely to consume unhealthy foods (OR = 1.32; 95% CI: 1.11–1.56), artificial juices or soft drinks (OR = 1.32; 95% CI: 1.08–1.61) and to replace meals with snacks (OR = 2.15; 95% CI: 1.38–3.34). Furthermore, night shift work was significantly associated with the habits of smoking (OR 1.32; 95% CI: 1.10–1.59) and alcohol consumption at least once a week (OR = 1.48; 95% CI: 1.23–1.78).

The associations of night shift work with exposure to possible carcinogens by gender are presented in Table 4. Among male night workers, the likelihood of handling of radioactive material and biological material were, respectively, 2.45 times (OR 2.45; 95% CI 1.61–3.72) and 3.54 times (OR = 3.54; 95% CI: 2.39–5.25) higher than among daytime shift workers. Among females, the likelihood of exposure to radioactive material (OR = 3.89; 95% CI: 3.15–4.80), chemical agents (OR = 1.54; 95% CI: 1.20–1.97), biologic material (OR = 3.89; 95% CI: 3.15–4.80) and passive smoking at work (OR = 1.45; 95% CI: 1.12–1.86) were statistically higher among night shift workers compared to day shift workers.

## 4. Discussion

Night shift work has increased substantially in recent decades and has been increasingly addressed in the international literature due to the associated risk of cancer, including breast, prostate, and colon and rectum cancer, which are the three most common types of cancer in Brazil.

Currently, the global proportion of night shift workers is considerable and merits further studies on the burden of diseases associated with night shift work. In Brazil, a 14% prevalence of night shift workers was found. Compared with other countries, similar prevalence estimates of night shift work were observed in the United States, 17% [9] and in European regions where the prevalence ranged from 14 to 21% [26]. However, the definition of night shift work varies across nations, in terms of the minimum duration of a night shift or the period of a night shift [26,27]. Regarding gender, the pattern of higher proportions of night shift work among men than among women was consistent with international studies [8,9,26].

In relation to the harmful effects on health, our results revealed that night shift workers had markedly higher prevalences of sleep disorders and tiredness compared to day shift workers, as well as depressive feelings among female night shift workers. Evidence of higher rates of insomnia, fatigue, exhaustion, and depressive feelings among night shift workers has been reported previously [11,12,28]. Differences by gender could be explained by the fact that most women who work at night are also responsible for household chores and family care, making them more vulnerable to emotional problems than men [29]. Furthermore, it is well-known that women report their health problems more than men [30].

Our study also revealed that night shift workers were more likely to have unhealthy eating patterns than day shift workers. Corroborating our results, findings of a systematic review with 31 observational studies showed that the eating behavior of rotational night shift workers differed from day shift workers with fixed working hours. The former presented irregular mealtimes, increased snacking/eating at night, lower consumption of healthy foods, such as fruits and vegetables and a higher intake of processed foods, such as soft drinks and sugary beverages [31].

Considering obesity, our findings indicated a significant positive association of obesity with night shift work among males. The higher consumption of unhealthy food and meal replacements with snacks reported by night shift workers are a likely explanation for this association. Another Brazilian study of 2372 nurses from 18 public hospitals in Rio de Janeiro showed that time on a night shift work was positively associated with BMI for both sexes, although this association was more pronounced among men than women [23]. Similarly, a study of 2059 nursing staff members in Norway [15] indicated that the number of hours worked at night was a factor associated with obesity, but it also affects men more than women. These results are of concern, as obesity is known to be associated with 11 types of cancer, in addition to being a risk factor for cardiovascular disease and other metabolic disorders [32]. A possible mechanism for the increased risk of obesity in night shift workers is the sleep–wake cycle modification, inducing short sleep periods that affect blood concentrations of hormones that regulate hunger and satiety [33]. These alterations in the circadian cycle result in excessive caloric intake during the night, which impairs glucose metabolism and lipid homeostasis, conditions that are associated with obesity [33].

The findings related to smoking also deserve attention as women who work at night are more likely to smoke regularly than those who work during the day, similarly to other survey results [33,34].

Regarding alcohol consumption, a significant and positive association with night shift work for males and females was found. Previous studies have also pointed to a higher risk of alcohol consumption among night shift workers [35,36,37]. The greater consumption of alcohol and tobacco among night shift workers is often justified as a way of mitigating tiredness and fatigue related to sleep disorders and psychological stress imposed by changes in the circadian cycle [38]. In fact, it is already known that alcohol is a psychoactive substance and can initially induce a feeling of pleasure, well-being, and relaxation when consumed in low doses [39]. However, in 1988, the IARC classified the consumption of alcoholic beverages as carcinogenic to humans (Group 1) for mouth, pharynx, larynx, esophagus, and liver cancers. Later reviews occurred in 2010, presenting new evidence for colon, rectal and breast cancer and, in 2012, for pancreatic and stomach cancer [6]. Evidence indicates that the risk of cancer is directly proportional to the amount of alcohol consumed and the time of exposure. The risk is even greater for those who use alcohol and tobacco at the same time, about 2 to 3 times higher compared to those who do not [40].

Regarding possible occupational exposure to carcinogens among workers, night work was positively associated with handling radioactive material for both sexes. A survey carried out in Australia [41] with 5,425 workers showed that prevalence of ionizing radiation was higher in night shift workers compared to day shift workers with fixed working hours. There is a wide variety of types of cancer related to radioactive material exposure, namely cancer of the salivary gland, esophagus, stomach, colon, lung, bone, breast, kidney, skin (basal cell), brain, thyroid, and leukemias [42]. Exposure of workers to radioactive material on the night shift is often reported in hospital sectors and diagnostic imaging laboratories, nuclear power plants, and activities in underground hematite mines [43].

Among females, passive smoking was significantly associated with night shift work. Exposure to passive smoking at work has been well-documented in the scientific literature. A Danish study with 5940 workers found a higher likelihood of passive smoking among night shift workers who worked two or more-night shifts [44]. It is important to highlight that passive smoking has sufficient evidence for lung cancer in humans and limited evidence for pharyngeal and laryngeal cancer [42], indicating that this group of workers are more vulnerable to these neoplasms.

In relation to chemical exposure, our findings showed that the chance of exposure to chemical substances was significantly higher among female night shift workers compared to day shift workers. The IARC has classified more than 100 chemical agents that are often used in sectors in which night shift work is frequent as carcinogenic to humans (group 1, group 2A, and group 2B). These sectors include health services, construction, iron and steel founding, hematite mining (underground), and aluminum production, among others [42]. Furthermore, occupational exposure to toxic chemical agents during the reproductive age, which was the predominant age group of Brazilian women who work at night, can cause endocrine disruption, early menopause, reduced fertility, menstrual cycle imbalance and adverse effects on pregnancy [45].

Additionally, higher chances of exposure to biological material were found for male and female night shift workers. Studies carried out on health professionals who worked at night in Saudi Arabia and Thailand presented similar results [46,47]. In regard to the association between exposure to biological agents and cancer, the scientific literature has shown that latent infections or prolonged recurrent infections with Human Papilloma Virus (HPV), Hepatitis B virus (HBV), Hepatitis C virus (HCV), and Helicobacter pylori are the main causes of cervical cancer, liver cancer, and stomach cancer, respectively, for which female sex workers [48] and healthcare workers [49] represent the most vulnerable occupational group.

The strength of this study is the use of a probabilistic and representative sample of the Brazilian employed population. Nevertheless, the PNS is not a specific survey designed to assess the working conditions of the Brazilian population. So, there is no detailed information on night shift schedules, whether they are rotative or not. In addition, questions related to sleep disorders and other mental health conditions are limited to those derived from the PHQ-9. Therefore, hours of sleep, changes in sleep patterns during weekends or rest periods, among others, are unknown.

A caveat of our investigation lies in the number of occupational carcinogens assessed by the PNS. Since it is not a specific survey on cancer, we were able to assess only four possible carcinogenic exposures among night shift workers (handling of chemicals, radioactive material, passive smoking at work, and biological agents). It should be added that exposure to handling chemical substances and biological material is collected in an aggregated manner, without identifying the type of chemical and biological agent. These variables could be considered as a proxy for exposure to chemical carcinogens and oncogenic viruses, by reflecting on the possible occupational categories that might be exposed to these agents.

Another restriction in our study is that the question refers only to the presence or absence of exposure in the worker’s current main occupation. Therefore, it is not possible to assess occupational exposure to multiple carcinogens throughout a person’s working life. Future studies focusing on investigation of simultaneous carcinogenic occupational exposures in night workers in Brazil should be conducted by specific surveys in Brazilian employed population.

It is also important to clarify that the number of hours per day and the number of days per week were not considered in this study, due to sample size issues.

## 5. Conclusions

This is the first time that national data from a representative sample of the Brazilian working population have been analyzed with a focus on night work and its possible health implications. Therefore, the present study reinforces the scientific evidence on the adverse health effects of night work among Brazilian workers, which are still largely unknown to the general population and health policymakers. The study’s findings can be used to support public policies aimed at protecting and promoting workers’ health in Brazil and the findings should be considered in managing and organizing night shift work in the workplace.

The results found in the present study should be considered in managing and organizing night shift work in the workplace. Worker health programs should be implemented in all public and private organizations to (1) promote safe and healthy working environments, (2) identify and manage occupational risks, and (3) permanently monitor workers’ health through clinical and laboratory tests. This program should focus on actions such as offering small healthy meals, rich in fruits, vegetables, and grains, and discouraging the intake of ultra-processed foods. Campaigns in the workplace to reduce passive smoking at work and the consumption of alcohol should also be conducted. Furthermore, educational programs about the use of individual protective measures and special retirement schemes for night shift workers must also be considered to reduce the exposure to occupational carcinogens.

## Figures and Tables

**Table 1:** Prevalence of night shift workers aged 18–59 years according to sociodemographic characteristics. Brazil, 2019.

Sociodemographic Characteristics	Total (n = 6599)			
	%	95% CI	OR*	*p*-Value
**Total**	**13.8**	**13.2–14.4**	**-**	**-**
**Gender**				
Male	17.0	16.1–18.0	1.90	*p* < 0.01
Female	9.7	9.0–10.5	1.00	-
**Age range**				
18 to 29 years	14.4	13.1–15.7	1.17	0.01
30 to 39 years	15.1	14.1–16.2	1.25	*p* < 0.01
40 to 59 years	12.5	11.7–13.3	1.00	-
**Skin color**				
White	13.9	13.0–14.9	1.00	-
Mixed	13.4	12.6–14.2	0.96	0.42
Black	14.6	13.1–16.3	1.06	0.42
**Level of Education**				
Incomplete primary education	10.1	9.2–11.1	1.00	-
Incomplete secondary education	12.7	11.4–14.2	1.30	*p* < 0.01
Incomplete college education	16.2	15.2–17.3	1.73	*p* < 0.01
College education or higher	13.7	12.6–14.8	1.41	*p* < 0.01
**Income per capita**				
Less than 1 MW	8.1	7.2–9.1	1.00	-
Between 1 and 2 MW	13.7	12.8–14.7	1.80	*p* < 0.01
More than 2 MW	17.4	16.4–18.5	2.39	*p* < 0.01
**Household situation**				
Urban	14.6	13.9–15.3	2.08	*p* < 0.01
Rural	7.6	6.8–8.5	1.00	-

Key: OR* = odds ratio and *p*-value estimated by bivariate logistic regression, MW: minimum wage.

**Table 2:** Associations of night shift work with selected health problems by gender. Brazil, 2019.

Health Problems	Night Shift Work				OR	95% CI	*p*-Value
	Yes		No				
	n	%	n	%			
**Male**							
Sleep disorders	626	13.9	2282	10.4	1.39	1.17–1.65	*p* < 0.01
Tiredness	614	13.6	1885	8.6	1.68	1.41–2.0	*p* < 0.01
Lack of concentration	221	4.9	881	4.0	1.24	0.91–1.68	0.18
Depressive feelings	224	5.0	884	4.0	1.25	0.95–1.63	0.11
Obesity	1077	21.2	3426	16.0	1.41	1.20–1.66	*p* < 0.01
**Female**							
Sleep disorders	470	22.4	3700	19.1	1.23	1.03–1.46	0.02
Tiredness	506	24.2	3847	19.8	1.29	1.08–1.54	0.01
Lack of concentration	197	9.4	1713	8.8	1.07	0.84–1.38	0.58
Depressive feelings	306	14.6	2187	11.3	1.35	1.09–1.67	*p* < 0.01
Obesity	452	11.0	1644	9.4	1.18	0.98–1.43	0.08

Key: OR = odds ratio and *p*-value estimated by bivariate logistic regression.

**Table 3:** Associations of night shift work with unhealthy behaviors by gender. Brazil, 2019.

Health Behaviors	Night Shift Work				OR	95% CI	*p*-Value
	Yes		No				
	n	%	n	%			
**Male**							
Regular consumption of unhealthy foods	1787	39.7	7440	33.9	1.28	1.12–1.46	*p* < 0.01
Regular consumption of artificial juices or soft drinks	1319	29.3	5523	25.2	1.23	1.07–1.41	*p* < 0.01
Often replacing meals with snacks	169	3.8	335	1.5	2.52	1.66–3.83	*p* < 0.01
Regular consumption of sweets	766	17.0	3134	14.3	1.23	1.04–1.45	0.02
Current smoker	729	16.2	3505	16.0	1.01	0.87–1.17	0.93
Alcohol consumption at least once a week	2004	44.5	9013	41.1	1.15	1.02–1.29	0.02
**Female**							
Regular consumption of unhealthy foods	781	37.3	6040	31.1	1.32	1.11–1.56	*p* < 0.01
Regular consumption of artificial juices or soft drinks	507	24.2	3785	19.5	1.32	1.08–1.61	0.01
Often replacing meals with snacks	115	5.5	513	2.6	2.15	1.38–3.34	*p* < 0.01
Regular consumption of sweets	406	19.4	3200	16.5	1.22	0.98–1.51	0.08
Current smoker	277	13.2	1708	8.8	1.32	1.10–1.59	*p* < 0.01
Alcohol consumption at least once a week	632	30.2	4377	22.5	1.48	1.23–1.78	*p* < 0.01

Key: OR = odds ratio and *p*-value estimated by bivariate logistic regression.

**Table 4:** Associations of night shift work with exposure to carcinogens. Brazil, 2019.

Exposure to Carcinogens	Night Shift Work				OR	95% CI	*p*-Value
	Yes		No				
	n	%	n	%			
**Male**							
Handling of radioactive material	120	2.7	244	1.1	2.45	1.61–3.72	*p* < 0.01
Handling of chemical substances	887	19.7	4150	18.9	1.05	0.90–1.23	0.53
Passive smoking at work	497	14.8	1773	12.9	1.17	0.93–1.46	0.17
Exposure to biologic material	372	8.3	604	2.8	3.18	2.45–4.12	*p* < 0.01
**Female**							
Handling of radioactive material	103	4.9	280	1.4	3.54	2.39–5.25	*p* < 0.01
Handling of chemical substances	241	11.5	1,514	7.8	1.54	1.20–1.97	*p* < 0.01
Passive smoking at work	206	11.0	1,340	7.9	1.45	1.12–1.86	*p* < 0.01
Exposure to biologic material	445	21.3	1,259	6.5	3.89	3.15–4.80	*p* < 0.01

Key: OR = odds ratio and *p*-value estimated by bivariate logistic regression.

## Data Availability

The microdata from the National Health Survey (PNS) analyzed in this study are publicly available on the IBGE website at the following link: https://www.ibge.gov.br/estatisticas/sociais/saude/29540-2013-pesquisa-nacional-de-saude.html?edicao=9177&t=microdados (accessed on 23 January 2019).
